# Qualitative Phytochemical Profiling and In Vitro Antioxidant Potential Evaluation of South African Momordica Balsamina Linn Fruit Pulp

**DOI:** 10.3390/life15010004

**Published:** 2024-12-24

**Authors:** Buang Matseke, Sipho Mapfumari, Mmamosheledi Mothibe

**Affiliations:** 1 Department of Pharmaceutical Sciences, School of Pharmacy, Sefako Makgatho Health Sciences University, P.O. Box 218, Pretoria 0208, South Africa; 2 Department of Physiology, School of Medicine, Sefako Makgatho Health Sciences University, P.O. Box 211, Pretoria 0208, South Africa; 3 Division of Pharmacology, Faculty of Pharmacy, Rhodes University, P.O. Box 94, Grahamstown 6139, South Africa; mamza.mothibe@ru.ac.za

**Keywords:** antioxidant, radical scavenging, fruit pulp, phytochemicals, bitter melon

## Abstract

*Momordica balsamina* Linn is a well-known African traditional herb due to its tremendous medicinal and nutritional properties. It is used worldwide for the treatment of different ailments and diseases. In the present study, the phytochemical and antioxidant activity of South African *M. balsamina* fruit pulp extracts was evaluated. The fruit pulp extracts were obtained by using the serial exhaustive extraction procedure using the solvents hexane, DCM, acetone, and methanol. The resulting extracts were subjected to different standard colorimetric tests for phytochemical analysis. The presence of compounds with antioxidant activity was determined using dot plot and TLC. The DPPH radical scavenging assay, hydrogen peroxide activity, and reducing power assay, coupled with linear regression, were employed to determine the quantity of antioxidants and their IC50. The results for qualitative phytochemical screening have shown that the fruit pulp contains alkaloids, cardiac glycosides, saponins, phenolic compounds, tannins, flavonoids, terpenoids, and steroids. All the extracts revealed the presence of antioxidant activity in both dot plot and TLC. Acetone extracts (0.279 mg/mL) showed the lowest IC50 compared to the standards gallic acid (0.4 mg/mL) and diosgenin (0.42 mg/mL). These findings confirmed that *M. balsamina* is very rich in phytochemical compounds and has strong antioxidant potential; therefore, it could be a potential source of drugs which in the future may serve the production of synthetically improved therapeutic agents.

## Simple Summary

There is currently an increase in the trend of medicinal plant usage in developing countries leading to an escalation of research on plants used for different medicinal purposes and those well-known globally for their biological properties. Various medicinal plants have been tested and proven to possess biological activities including antibacterial, antidiabetic, anti-platelets, anti-inflammatory, antioxidant, anti-HIV, anti-diarrheal, antimicrobial, anti-cancer, and many more. One of the most important biological activities a plant can elicit is antioxidant because natural antioxidants from plants are responsible for the reduction of oxidative stress in the human body which is linked to the reduction of heart diseases, arthritis, cancer, stroke, and other inflammatory conditions. This study sought to establish the antioxidant potential of the bitter melon herb. It has a number of compounds which are known to be responsible for the treatment of a number of diseases and ailments such as alkaloids, phenolic compounds, and terpenoids. Furthermore, the fruits have been found to be a very strong antioxidant agent. This explains the usage of this plant in the management of hypertension and diabetes by indigenous communities.

## 1. Introduction

Since time immemorial, people have depended on plants as their source of medicine or treatment for various diseases. In as much as these plants have been used for that long, they were being used without being scientifically verified or documented formally [1]. Since the turn of the century, plants which are considered medically important are those which have been reported to contain several bioactive compounds that are responsible for the plant’s pharmacological properties [2,3]. These bioactive compounds are mainly secondary metabolites which, unlike primary metabolites, are produced for the protection of the plant against pathogens, UV radiation, and other environmental factors [3,4,5,6]. The compounds do, however, have important characteristics which elicit physiological responses that are pharmacologically significant in the alleviation and treatment of different types of diseases [7,8]. Amongst the well-studied of these compounds are alkaloids, flavonoids, glycosides, phenolic acids saponins, tannins, and terpenes [9,10,11,12]. Recent laboratory in vitro studies have scientifically linked these phytochemicals to several biologically important activities such as anti-fungal, antidiabetic, anti-cancer, anti-bacterial, and antioxidant activity [13,14,15].

Antioxidants, also called free radical scavengers, are compounds that have the ability to protect cells either by preventing or reducing the oxidative damage resulting from accumulation of free radicals [16,17]. Free radicals are molecular species with very high reactivity and are produced as by-products of numerous physiological and biochemical processes in the body [18]. It is reported that they are generated by some specialized enzymes such as nitric oxide synthase, myeloperoxidase, and NADPH-oxidase [19,20]. At normal concentrations, free radicals play a role in the maturation process of cellular structures and in the host defence system [21]. Free radicals released by neutrophils, monocytes, and macrophages help to fight against pathogenic microbes that cause diseases [22,23]. Excessive production of these free radicals induces oxidative stress, which is associated with many conditions such as diabetes, cancer, inflammation, ischemic heart disease, atherosclerosis, hair loss, immunosuppression, Alzheimer’s disease, and Parkinson’s disease [24,25,26]. In order to maintain oxidants within levels which are not physiologically harmful, either synthetic or natural antioxidants can be administered to reduce the activity of these free radicals either by suppressing their formation or by counteracting their actions [27,28]. Synthetic antioxidants have, however, been reported to be associated with carcinogenesis and liver damage; those from natural sources and plants, in contrast, appear to serve as safer alternatives [29,30].

Several studies have reported on the potential of medicinal plants to be antioxidant agents [31,32]. Such plants include but are not limited to *Aloe ferox*, *Moringa oleifera*, *Hypoxis hemerocallida*, *Artemisia afra*, *Harpagophytum procubens*, and *Momordica balsamina* [33,34,35,36,37,38,39]. *M. balsamina* Linn is one of the plants that have been studied worldwide for their nutritional and medicinal properties [40,41,42]. It is said to be used in the treatment of different ailments and diseases such as diabetes, malaria, diarrhoea, and skin disorders such as scabies, and it has other biological activities such as antimicrobial, anti-HIV, anti-bacterial, and antioxidant activity [43,44,45,46,47]. This species is also utilized by some South African indigenous knowledge practitioners, where it has been recently reported that about 60–80% of the population use or depend on medicinal plants or their concoctions to treat various ailments [48,49,50]. The current study therefore reports on the outcomes of the investigation of the phytochemicals as well as the antioxidant properties of indigenous *M. balsamina* Linn fruit pulp collected from Mpumalanga, South Africa.

## 2. Materials and Methods

### 2.1. Plant Material

Sample collection, preparation, and handling

Fresh fruits of *M. balsamina* were collected from Mpumalanga province in a village known as Phake ya Ratlhagane (25°08′51.1″ S 28°30′28.2″ E) with the assistance of a traditional medicine practitioner. A fresh sample was taken to the South African National Biodiversity Institute (SANBI) for botanical identification. The fruits were cut into halves and left to air dry and the seeds were then taken out. The dried fruits were ground into a fine powder using a Polymix Laboratory Dry Mill Drive Unit (Polymix™ PX-MFC 90 D, Kinematica AG, Luzern, Switzerland) and then stored at room temperature in the laboratory cupboard for further use.

Extraction of the plant material

The powdered sample (449.52 g) was extracted following a serial exhaustive extraction method, and the solvents used were hexane, dichloromethane (DCM), acetone, and methanol starting from the least polar to the most polar solvent. The extraction was carried out with 1.5 L of each solvent on an orbital platform shaker (Model 261, 8 kg, 230–50 Hz, 120 W, Labotec, South Africa). This process was carried out in a 24 h cycle and repeated three times for each solvent. The resultant supernatants were filtered using filter discs (Filter Discs Qual. 3 hw, 125 mm, BOECO, Hamburg, Germany). The filtrate was concentrated using a Stuart rotary evaporator (RE400, Cole-Parmer Ltd. Stone, ST15 OSA, Staffordshire, UK) at a temperature of 37 °C with a speed of 50 cycles per minute and the final evaporation of the solvents took place under a stream of air in the laboratory. The resultant extracts were then stored at room temperature in the laboratory cupboard for further use.

### 2.2. Qualitative Phytochemical Analysis

The presence of phytochemicals in *M. balsamina* fruit pulp extracts was analysed using the standard colometric tests as reported by [51,52,53] with minor modifications where all the extracts were dissolved in the solvents n-hexane, DCM, acetone, and methanol. Each extract was dissolved in a solvent that was used to extract them prior to the standard colorimetric tests as follows:

Test for alkaloids

The Dragendorff reagent (potassium-bismuth-iodide solution) test was used to determine the presence of alkaloids in the plant extracts. An amount of 3 mL of the extract was added into a test tube and to that extract, 15 drops of Dragendorff reagent were added continuously. The formation of a reddish-brown to orange precipitate in the test tube was taken as an indication of the presence of alkaloids in *M. balsamina* fruit pulp extracts.

Test for anthraquinones

To test for the presence of anthraquinones in the plant extracts, 1 mL of the extract was treated with 20 drops of the prepared 10% ammonia solution. The appearance of the pink-coloured precipitate would have been an indication of the existence of anthraquinones in the plant extract.

Test for cardiac glycosides

To determine the presence of cardiac glycosides in the extracts, the crude extract (2 mL) was treated with 1 mL of glacial acetic acid, 1 mL of ferric chloride, and 1 mL of concentrated sulphuric acid (H_2_SO_4_). The occurrence of the green-blue colour of the solution denoted the presence of cardiac glycosides in the plant extracts.

Test for saponins

The crude extract (0.5 mL) was mixed with 5 mL of distilled water and then shaken vigorously to determine the presence of saponins in the extracts. The formation of foam and persistence of frothing were evidence of the presence of saponins.

Test for phenolic compounds

The ferric chloride test was used to determine the presence of phenolic compounds in the plant of study. An aliquot of 2 mL of the crude extract was mixed with 0.5 mL of ferric chloride. The intense brown and reddish colour that appeared in the mixture was taken as an indication of the presence of phenolic compounds.

Test for flavonoids

For the confirmation of flavonoids in *M. balsamina* extracts, 2 mL of the crude extract was mixed with 1 mL of dilute NaOH. This was followed by the addition of 1 mL of dilute HCl. A yellowish colour that appeared after the addition of NaOH indicated the presence of flavonoids, and a colourless solution that appeared after the addition of dilute HCl confirmed the presence of flavonoids.

Test for tannins

To test for the presence of tannins in the extracts, 1 mL of ferric chloride was added to 2 mL of the crude extract. The occurrence of green, blue, and brown or blue-black showed positive results for tannins in the solution.

Test for terpenoids

To test for the presence of terpenoids, an amount of 5 mL of the extract was added into a test tube followed by the addition of 2 mL of chloroform and sidewise addition of 3 mL of H_2_SO_4_. The formation of the reddish-brown colour of the interface confirmed the presence of terpenoids in the plant extract.

Test for steroids

The presence of steroids was determined by the addition of 1 mL of chloroform and a sidewise addition of 1 mL of concentrated sulphuric acid to 2 mL of the crude extract. The appearance of a red colour in the lower chloroform layer represented the presence of steroids in the plant extracts.

### 2.3. Antioxidant Activity

#### 2.3.1. Qualitative Antioxidant Activity

Dot plot method

The stored extracts of *M. balsamina* were redissolved in the solvents that were used to extract them. A pre-coated silica gel 60 thin layer chromatography (TLC) plate with fluorescent indicator UV_254_ was prepared in such a way that it accommodated the plotting of all four extracts (5 cm × 10 cm). Each extract was spotted in a dot form on the TLC plate, and the plate was then left to dry under a stream of air in the laboratory for 5 min. The TLC plate was then sprayed with the prepared 0.2 mM of 2,2-diphenyl-1-picrylhydrazyl (DPPH) solution. The white to pale yellow discoloration on the purple background of the TLC plate was taken as an indication of antioxidant activity.

TLC antioxidant screening

The dried extracts were redissolved in the solvents that were used to extract them. The pre-coated silica gel 60 TLC plates with fluorescent indicator UV_254_ were prepared for each extract (5 cm × 10 cm). The extracts were spotted on a baseline that had been drawn at 1.5 cm from the bottom of the plate. Solvent systems of different polarities were used as mobile phases to develop the TLC chromatograms of the extracts. Hexane was developed with hexane: ethyl acetate (8:2), DCM with hexane: ethyl acetate (9:1), acetone and methanol with chloroform: ethyl acetate: formic acid (4.9:3.9:0.5). Once the solvent front had reached about 80% of the TLC plates, the plates were taken out of the TLC chamber and a line was drawn at that point. The developed TLC plates were observed under normal light, UV short wave (214 nm), and UV long wave (314 nm). The TLC plates were then sprayed with DPPH solution and allowed to dry. The appearance of pale yellow to white discoloration of purple DPPH indicated the presence of antioxidant activity in the plant extracts.

#### 2.3.2. Quantitative Antioxidant Activity Analysis

DPPH antioxidant activity assay

The DPPH radical scavenging activity of the plant extracts was determined following the methods described by [52,54] with a few modifications. Standard solutions of various concentrations of the extracts (0.2, 0.4, 0.6, 0.8, and 1 mg/mL of the extracts), as well as 0.8 mM of DPPH in methanol, were prepared. Then 1 mL of each prepared standard was transferred into a test tube and an amount of 1 mL DPPH solution was then added. The mixture was vortexed and then incubated for 30 min at room temperature. Thereafter, 200 µL of each of the mixtures were transferred into separate wells of a 96 well plate, and their absorbances were measured at 517 nm against the blank. Ascorbic acid and butylated hydroxytoluene (BHT) were used as positive control standards. The ability of the plant extracts to scavenge the free radical was calculated using Equation (1) below for each extract.

(1) $$\%\;DPPH\;radical\;scavenging\;activity=\frac{A_{0}-A_{S}}{A_{0}}\times 100$$

where:

*A_s_* = absorbance of the sample

*A*_0_ = absorbance of negative control

Hydrogen peroxide activity assay

A hydrogen peroxide (H_2_O_2_) activity assay described by [52,53] with minor modifications was used to determine the ability of the plant extracts to scavenge hydrogen peroxide. Different concentration standards (0.2, 0.4, 0.6, 0.8, and 1.0 mg/mL) of the extracts were prepared and an amount of 1 mL of each standard was transferred into a test tube. To each of the extracts, 2 mL of a solution of 40 mM and phosphate buffer (pH 7.4) was added. The reaction mixture was vortexed at 3000 rpm and then incubated for 10 min at room temperature. An amount of 200 µL of each mixture was transferred into a well on a 96 well plate, and their absorbances were measured at 560 nm against blank. Gallic acid, BHT, and diosgenin were used as control standards, respectively. The ability of the extracts to scavenge the H_2_O_2_ was calculated using Equation (2) below.

(2) $$H_{2}O_{2}\;scavenging\;activity\;\%=\frac{A_{0}-A_{S}}{A_{0}}\times 100$$

where

$A_{S}$ = Absorbance of sample

*A*_0_ = Absorbance of negative control

Reducing power assay

To determine the reducing power ability of the plant extracts, a method described by [52,53] was followed with minor modifications. *M. balsamina* extracts were redissolved in the solvents used to extract them (1 mg/mL). Different concentration standards (0.2, 0.4, 0.6, 0.8, and 1 mg/mL) of the extracts were prepared. An amount of 1 mL of each concentration was transferred into a test tube. To that extract, 2.5 mL of 1% potassium ferricyanide (K_3_Fe(CN)_6_) and 2.5 mL of 0.2 M sodium phosphate buffer (pH 6.6) were added. The reaction mixture was vortexed at 3000 rpm and then incubated at 50 °C for 30 min. Following that, 2.5 mL of 10% trichloroacetic acid (TCA) was added and centrifuged at 3000 rpm for 10 min. An amount of 2.5 mL of the supernatant was mixed with 2.5 mL of deionized water and 0.5 mL of 0.1% ferric chloride. The absorbances of each mixture were measured at 700 nm against blank. BHT, gallic acid, and diosgenin were used as standards. The reducing power ability of the samples were determined using Equation (3) below.

(3) $$Reducing\;power\;activity\%=\frac{A_{0}-A_{S}}{A_{0}}\times 100$$

where

$A_{S}$ = Absorbance of sample

*A*_0_ = Absorbance of negative control

## 3. Results

### 3.1. Qualitative Phytochemical Analysis

The four extracts of *M. balsamina* were subjected to the standard chemical reaction tests for phytochemical screening. The full phytochemical qualitative profile results are reported in Table 1 below:

The qualitative phytochemical results as shown on Table 1, indicated the presence of a variety of phytochemicals in the different *M. balsamina* pulp’s extracts. Alkaloids were found to be moderately present in the hexane extracts, present in traces in the DCM extract, while absent in both acetone and methanol. The test used for anthraquinones did not indicate their presence across all the extracts. Cardiac glycosides were only detected to be present in the DCM extract. Saponins and phenolic compounds were similarly present across the four extracts. Tannins were found to be present in the more polar extracts, while flavonoids were detected in the least polar extracts. This study found that terpenoids are better extracted as the polarity of the used solvents move from least polar to most polar. To this end, they were detected as traces in the DCM extract, moderately present in the acetone extract, and impressively present in the methanol extract. Unsurprisingly, the steroids were detected on both hexane and acetone which are the nonpolar solvents used in this study.

### 3.2. Antioxidant Activity

#### 3.2.1. Qualitative Antioxidant Activity

A solution of DPPH dissolved in methanol was used as an indicator, and the formation of a creamy white to pale yellow spots/bands against the purple background on the TLC plates indicated the presence of antioxidants in the extracts. The Rf values of the bands were calculated and recorded in Table 2.

Dot plot

**Figure 1:**
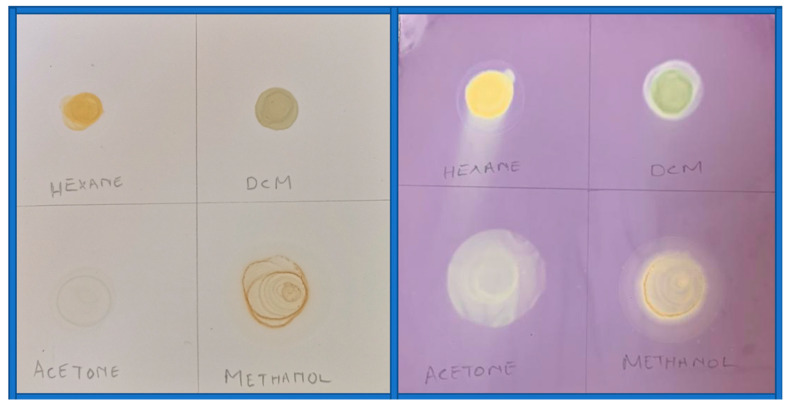
Dot plot of *M. balsamina* extracts before spraying with DPPH (**left**) and after spraying with DPPH (**right**).

**Figure 2:**
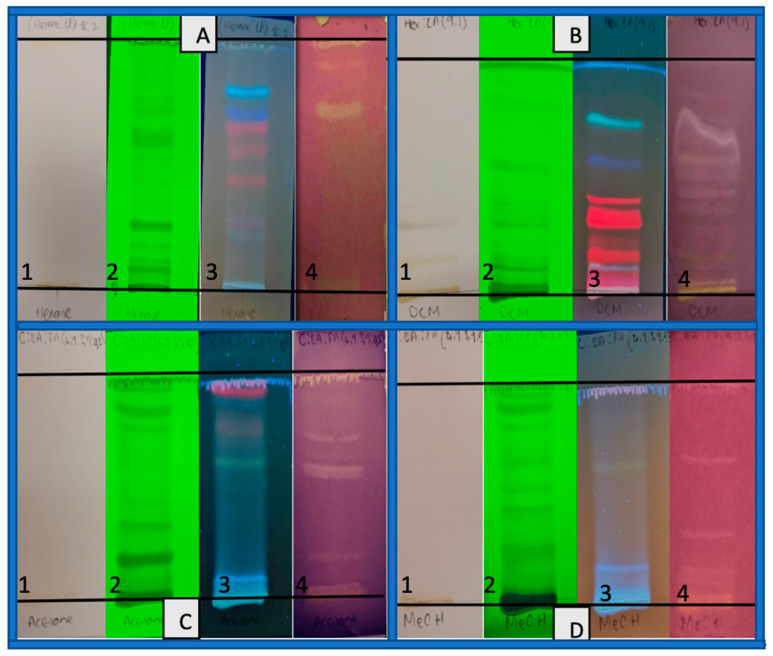
Chromatograms of *M. balsamina* (**A**) hexane, (**B**) DCM, (**C**) acetone and (**D**) methanol extracts developed with different solvent systems. The chromatograms are visualised from left to right under (1). visible light, (2). UV short wave (214 nm), (3). UV long wave (314 nm), and (4). DPPH spray for antioxidants.

The dot plot as depicted in Figure 1 showed that all the crude *M. balsamina* extracts were capable of acting as antioxidant agents. This meant that in each of the extracts there were one or more compounds that acted as antioxidants; however, these dot plot results do not provide us with information on whether this activity observed is a function of synergism or not. To assess this, developed TLC plates of all the extracts were performed and are represented by Figure 2.

**Table 2:** Rf values for the antioxidant activity bands as calculated from Figure 2.

	*M. balsamina* Extracts			
	n-Hexane	DCM	Acetone	Methanol
Rf Values of compounds with antioxidant activity	0.29 0.11	0.19 0.30 0.42 0.46 0.54 0.62 0.70 0.82 0.86	0.29 0.37 0.42 0.46 0.77 0.87	0.36 0.66 0,78 0.82 0.87
Total	2	9	6	5

TLC antioxidant screening

The hexane TLC plate [A4] showed two bands with Rf values of 0.29 and 0.11 which were positive for antioxidant activity. A dark blue band (Rf = 0.29) under long wave (A3) which has been reported to represent either phenolic acids or anthocyanidins can be clearly observed. This band corresponded with an antioxidant band that appeared after spraying with DPPH as seen at [A4]. Across all the extracts tested, the DCM extract had the highest number of bands positive for antioxidant activity (Figure 2, [B4]). These bands correspond to the Rf values of 0.19, 0.30, 0.42, 0.46, 0.54, 0.62, 0.70, 0.82, and 0.86 as can be observed form Figure 2 [B4]. A light blue band in [B3] that corresponds with a band in [B4] can be an indication of terpenoids [54].

The acetone extract [C4] showed six bands with antioxidant activity at Rf values of 0.29, 0.37, 0.42, 0.46, 0.77, and 0.87. The methanol extract [D4] at Rfs of 0.36, 0.66, 0.78, 0.82, and 0.87 showed about five bands with antioxidant activity. The yellowish band that appeared in [C3] and [D3] corresponding with the antioxidant bands in [C4] and [D4] might be an indication that the antioxidants of the two extracts are due the presence of flavonoids which are known to give off a yellow colour under UV light. This therefore suggests that the strength of antioxidant activity of the hexane, DCM, acetone, and methanol extracts as reported in Figure 1 on the dot plot may be as a result of combined synergistic effect of the compounds within each of the crude. The antioxidant activity of *M. balsamina* pulp extracts may be as the result of the presence of phenolic compounds, terpenoids, and flavonoids across the extracts as reported in Table 1.

#### 3.2.2. Quantitative Antioxidant Activity

The ability of the different extracts of *M. balsamina* to quantitatively neutralize oxidants was evaluated using the DPPH assay, hydrogen peroxide (H_2_O_2_) assay, and reducing power activity assay. The extracts were compared to the standards butylated hydroxytoluene (BHT) and gallic acid (GA). The IC50 values were then determined using the linear regression method from the plot obtained from different concentrations of each fraction.

DPPH radical scavenging assay

**Figure 3:**
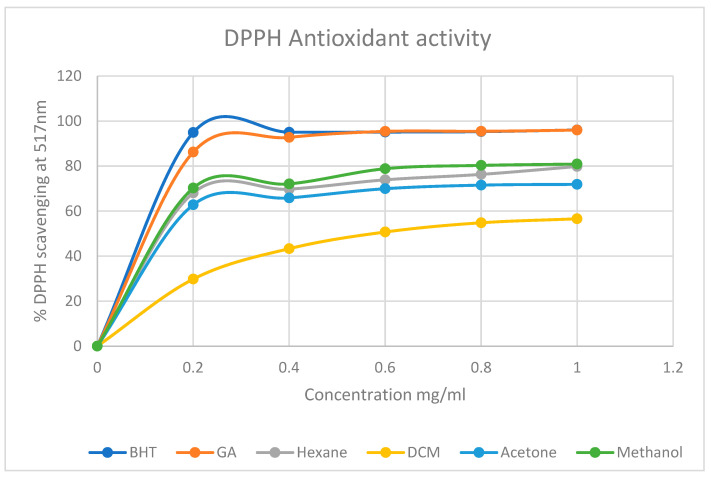
Percentage DPPH radical scavenging activity of *M. balsamina* extracts of different concentrations.

The results as depicted in Figure 3 concurred with the dot plot and the qualitative results and confirmed that all the extracts of *M. balsamina* are wholly capable of acting as antioxidant agents. Their strength and activity, however, appeared to be concentration but not polarity dependent. That is, the higher the concentration of the extract, the more the DPPH scavenged. Furthermore, the least polar solvent of extraction used (n-hexane) and the most polar (methanol) of the solvents used for extraction appeared to have similar DPPH scavenging activity trends and percentage inhibition activity with the highest for both being 79.83 and 80.91%, respectively. The DPPH scavenging activity trends for all the extracts were similar to that of the two standards used albeit with lower values.

Hydrogen peroxide scavenging activity assay

**Figure 4:**
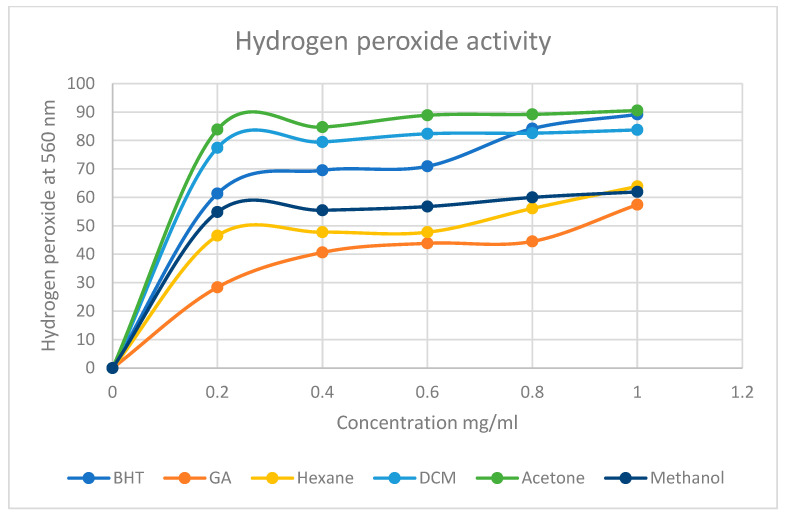
Percentage hydrogen peroxide radical scavenging activity of *M. balsamina* extracts of different concentrations.

In addition to their DPPH scavenging activity, *M. balsamina* extracts also have hydrogen peroxide (H_2_O_2_) scavenging activity, as depicted in Figure 4. The extracts followed the same trend as the standards where the H_2_O_2_ scavenging activity increases with the increase in concentration. At the highest concentration used, acetone extract showed the highest activity with 90.65%, and methanol extract showed the least activity (61.94%). Although the extracts have the same trend with standards, they seemingly are better scavengers of H_2_O_2_ than the standards. The high percentage scavenging activity of acetone may be as a result of synergistic action of multiple compounds found within the crude acetone extract.

Reducing power assay

**Figure 5:**
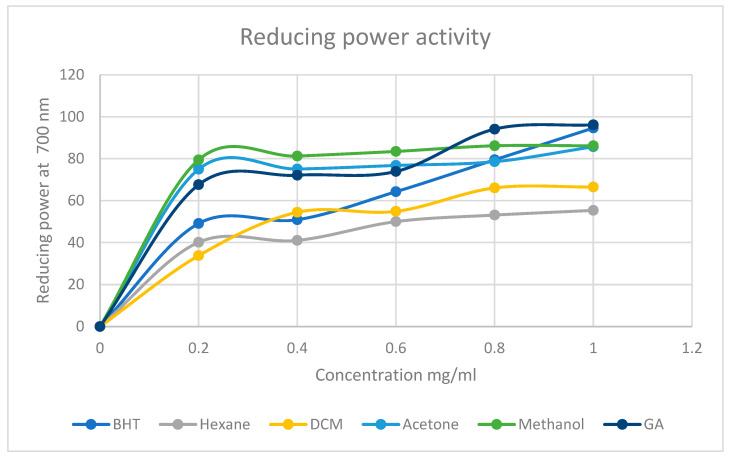
Percentage reducing power of *M. balsamina* extracts of different concentrations.

The results as depicted in Figure 5 indicate that all the crude extracts of *M. balsamina* have the reducing power ability. The reducing power of all the extracts followed similar trend as the standards where the reducing power increases as the concentration of the extracts increases. The activity of the extracts appears to be polarity dependent as it can be seen that at the highest concentration of 1 mg/mL, methanol exhibited the highest activity with a percentage of 86.16%, followed by acetone (85.71%), DCM (66.52%), and then hexane with 55.36%, although, as expected, the standards show the highest percentage of reducing power activity at 1 mg/mL, being 96.64% and 96.13%, respectively.

The graphs represented by Figure 3, Figure 4 and Figure 5 were used to determine the IC50 values of *M. balsamina* extracts. This was done using the linear regression method where the linear equation was drawn to best represent the data points as obtained using different concentration for each extract. The obtained IC50s were recorded on Table 3 above which shows that on average, across the three assays, the hexane extract had an IC50 of 0.571, DCM 0.49, acetone 0.279 and methanol 0.340. These IC50 values place the acetone extract as having much stronger antioxidant potential compared to the other extracts since it has the lowest average IC50. This implies that very little of the acetone extract is required to reduce oxidants. These are encouraging results as they suggest that traditional medicine practitioners who use convenient polar solvents for the preparation of concoctions benefit from the high antioxidant potential of the plant.

## 4. Discussion

The composition and quantity of phytochemical compounds in a plant might vary due to environmental factors that include seasonal variation, geographical location of growth, the growing stage, and environmental factors such as temperature, salinity, radiation, water, and many others [4,5,55,56]. The qualitative phytochemical profiling results of this study indicating the presence of a variety of phytochemicals in *M. balsamina* extracts, namely alkaloids, cardiac glycosides, saponins, phenolic compounds, tannins, flavonoids, saponins, and steroids, are of great importance given the pharmacological benefits these compounds offer. The presence of these compounds may also explain some of the traditional uses of this plant by different communities. Alkaloids are reported to be antimalaria agents, and this study confirming the presence of alkaloids in the extracts may be one of the plausible explanations for its usage in villages in Zimbabwe for the treatment and management for this communicable infection [57,58]. Given their known role in facilitating wound healing processes, the presence of saponins, flavonoids, and tannins within the plant fruit extracts might be responsible for the wound healing properties some communities associate with the *M. balsamina* plant [11,59,60,61,62]. Besides their diverse medical benefits, this study did not detect anthraquinones across all extracts, which is similar to the findings made and reported in previous studies [41,46].

Reactive oxygen species (oxidants) are known to have a detrimental effect on the physiology of both animals and humans, leading to the causation of various ailments and diseases such as cancer, diabetes, and cardiovascular diseases, amongst others [21,27,63], and the long-term usage of synthetic antioxidants is associated with gastrointestinal tract problems, skin allergies, and other health issues, which may include increased risk of developing cancer [16,26,30,64]. The importance of natural antioxidant medicine and human health can never be overstated. This is because studies have suggested that besides their role as antioxidants and while performing their role, they generate other bioactive molecules which are essential for the management as well as the treatment of other ailments and medical conditions [17,28,52,63,65]. As such, the results of this study have established that strong antioxidants within the fruit pulp of *M. balsamina* could serve as a plausible explanation for the ethnopharmacological usage of this plant by different communities and tribes. In South Africa to be specific, *M. balsamina* is used to treat liver diseases, treat vomiting associated with bile and fever, stomach, and intestinal complaints, and stabilize glycemia levels in diabetic patients [41,46,65,66]. This multifarious usage of *M. balsamina* in South Africa can only be linked to the observed strong antioxidant activity and the presence of the various phytochemicals identified in this study.

## 5. Conclusions

The current study has established *M. balsamina* to be rich in phytochemicals with significant antioxidant activity, thereby supporting its tremendous traditional usage throughout the world. Given the identification of these phytochemicals within the fruit pulp of this plant, the results suggest that *M. balsamina* could be a promising source of bioactive natural products with great pharmaceutical potential. The antioxidant activity of *M. balsamina* extracts may be a strong contributing factor to its applications in the management and treatment of diseases such as diabetes and high blood pressure. Further studies including isolation and identification of the target compounds responsible for the biological properties using the appropriate methods should be conducted to further explore its potential.

## Figures and Tables

**Table 1:** Results for qualitative phytochemical evaluation of *M. balsamina* fruit pulp extracts.

Phytochemicals Evaluated	Extracts of Differing Polarities			
	Hexane	DCM	Acetone	Methanol
Alkaloids	+	+/−	−	−
Anthraquinones	−	−	−	−
Cardiac glycoside	−	+	−	−
Saponins	+/−	+	+	++
Phenolic Compounds	+/−	+	+	++
Tannins	−	−	+	++
Flavonoids	+	+	−	−
Terpenoids	−	+/−	+	+++
Steroids	+	++	−	−

−: Absence; +/−: Trace Presence; +: Moderate presence; ++: Present in appreciable quantity; +++: Impressive presence.

**Table 3:** The IC50 values of various extracts of *M. balsamina* and standards BHT, gallic acid, and diosgenin.

Extracts and Standards	IC50 (mg/mL)		
	DPPH Scavenging	H_2_O_2_ Scavenging	Reducing Power
Hexane	0.315	0.672	0.726
DCM	0.701	0.218	0.551
Acetone	0.374	**0.206**	0.258
Methanol	**0.282**	0.539	**0.200**
BHT	0.072	0.330	0.422
Gallic acid	0.121	0.795	0.284
Diosgenin	ND	0.594	0.319

## Data Availability

All data for this study have been included in this manuscript, and any additional data can requested from the corresponding authors.
